# A Blueprint to Evaluate One Health

**DOI:** 10.3389/fpubh.2017.00020

**Published:** 2017-02-16

**Authors:** Simon R. Rüegg, Barry J. McMahon, Barbara Häsler, Roberto Esposito, Liza Rosenbaum Nielsen, Chinwe Ifejika Speranza, Timothy Ehlinger, Marisa Peyre, Maurizio Aragrande, Jakob Zinsstag, Philip Davies, Andrei Daniel Mihalca, Sandra C. Buttigieg, Jonathan Rushton, Luís P. Carmo, Daniele De Meneghi, Massimo Canali, Maria E. Filippitzi, Flavie Luce Goutard, Vlatko Ilieski, Dragan Milićević, Helen O’Shea, Miroslav Radeski, Richard Kock, Anthony Staines, Ann Lindberg

**Affiliations:** ^1^Vetsuisse-Faculty, Section for Veterinary Epidemiology, University of Zurich, Zurich, Switzerland; ^2^UCD School of Agriculture and Food Science, University College Dublin, Dublin, Ireland; ^3^Royal Veterinary College, London, UK; ^4^National Institute of Health, Rome, Italy; ^5^Faculty of Health and Medical Science, University of Copenhagen, Copenhagen, Denmark; ^6^Institute of Geography and Centre for Development and Environment, University of Bern, Bern, Switzerland; ^7^University of Wisconsin, Milwaukee, USA; ^8^CIRAD, Montpellier, France; ^9^Agriculture and Food Science Department, University of Bologna, Bologna, Italy; ^10^Swiss Tropical and Public Health Institute, University of Basel, Basel, Switzerland; ^11^Retired from 3ie, London, UK; ^12^Department of Parasitology and Parasitic Diseases, University of Agricultural Sciences and Veterinary Medicine, Cluj Napoca, Romania; ^13^Faculty of Health Sciences, University of Malta, Msida, Malta; ^14^Faculty of Health and Life Sciences, University of Liverpool, Liverpool, UK; ^15^Veterinary Public Health Institute, University of Bern, Bern, Switzerland; ^16^Department of Veterinary Sciences, University of Turin, Grugliasco-Turin, Italy; ^17^Veterinary Epidemiology Unit, Faculty of Veterinary Medicine, Ghent University, Ghent, Belgium; ^18^Faculty of Veterinary Medicine, Ss Cyril and Methodius University, Skopje, Macedonia; ^19^Institute of Meat Hygiene and Technology, Belgrade, Serbia; ^20^Cork Institute of Technology, Cork, Ireland; ^21^School of Nursing & Human Sciences, Dublin City University, Dublin, Ireland; ^22^National Veterinary Institute, Uppsala, Sweden

**Keywords:** One Health, evaluation criteria, sustainability, integrated approaches to health, evaluation framework, performance monitoring

## Abstract

One Health (OH) positions health professionals as agents for change and provides a platform to manage determinants of health that are often not comprehensively captured in medicine or public health alone. However, due to the organization of societies and disciplines, and the sectoral allocation of resources, the development of transdisciplinary approaches requires effort and perseverance. Therefore, there is a need to provide evidence on the added value of OH for governments, researchers, funding bodies, and stakeholders. This paper outlines a conceptual framework of what OH approaches can encompass and the added values they can provide. The framework was developed during a workshop conducted by the “Network for Evaluation of One Health,” an Action funded by the European Cooperation in Science and Technology. By systematically describing the various aspects of OH, we provide the basis for measuring and monitoring the integration of disciplines, sectors, and stakeholders in health initiatives. The framework identifies the social, economic, and environmental drivers leading to integrated approaches to health and illustrates how these evoke characteristic OH operations, i.e., thinking, planning, and working, and require supporting infrastructures to allow learning, sharing, and systemic organization. It also describes the OH outcomes (i.e., sustainability, health and welfare, interspecies equity and stewardship, effectiveness, and efficiency), which are not possible to obtain through sectoral approaches alone, and their alignment with aspects of sustainable development based on society, environment, and economy.

## Introduction

One Health (OH) positions health professionals as agents for change and provides a platform to both measure and manage determinants of health seldom fully covered by medicine or public health alone. The integration of human, animal, and environmental health has a long history (1–4). Recent financial, economic, social, environmental, and health crises have led to the renewed recognition that collaborative approaches between disciplines are urgently needed (5, 6). The fear of emerging pandemics, as well as climate change, drug resistance, food and water security and safety, has caused a shift from an interdisciplinary approach, whereby experts collaborate across disciplinary boundaries, to a transdisciplinary approach that integrates society and science by including all stakeholders (5, 7, 8). This transcends traditional boundaries, and integrates knowledge and perspectives from scientific and non-scientific sources (9, 10). Many communities involved in health issues have proposed transdisciplinary and systemic approaches with different focuses, such as Ecohealth, Global Health, Planetary Health, or Health in scaled Social–Ecological Systems (7, 8). While there is considerable literature describing what integrated approaches to health could be, there are no recognized guidelines—to our knowledge—on how to evaluate to what extent the underlying integration as a principle and approach contributes to constructive management of complex health problems, such as antibiotic resistance or outbreaks of highly infectious diseases, e.g., highly pathogenic avian influenza, Ebola, severe acute respiratory syndrome, and Zika virus disease. OH emphasizes the commonalities of human, animal, plant, and environmental health. In this perspective, it can be regarded as an “umbrella” term that captures integrative approaches to health across these highly interlinked components (4, 11). Due to the existing, historically contingent, organization of societies and disciplines, and the sectoral allocation of resources, developing integrated approaches is difficult, and benefits can be delayed. There is thus a need to provide evidence on the added value of OH to governments, researchers, funding bodies, and stakeholders (5, 12) and to explore how to evaluate integrated approaches to health. The Network for Evaluation of One Health (NEOH)^1^ is an initiative funded by the European Cooperation in Science and Technology that aims to address this by developing a framework and protocols for the evaluation of OH initiatives and by providing examples of their application.

The purpose of this paper is to identify and describe evaluable characteristics of OH approaches, and to present what they can encompass and achieve. This provides a basis for evaluation of OH initiatives and their outcomes, which could not be achieved using standard, sectoral approaches.

## Characterizing OH

**Figure 1:**
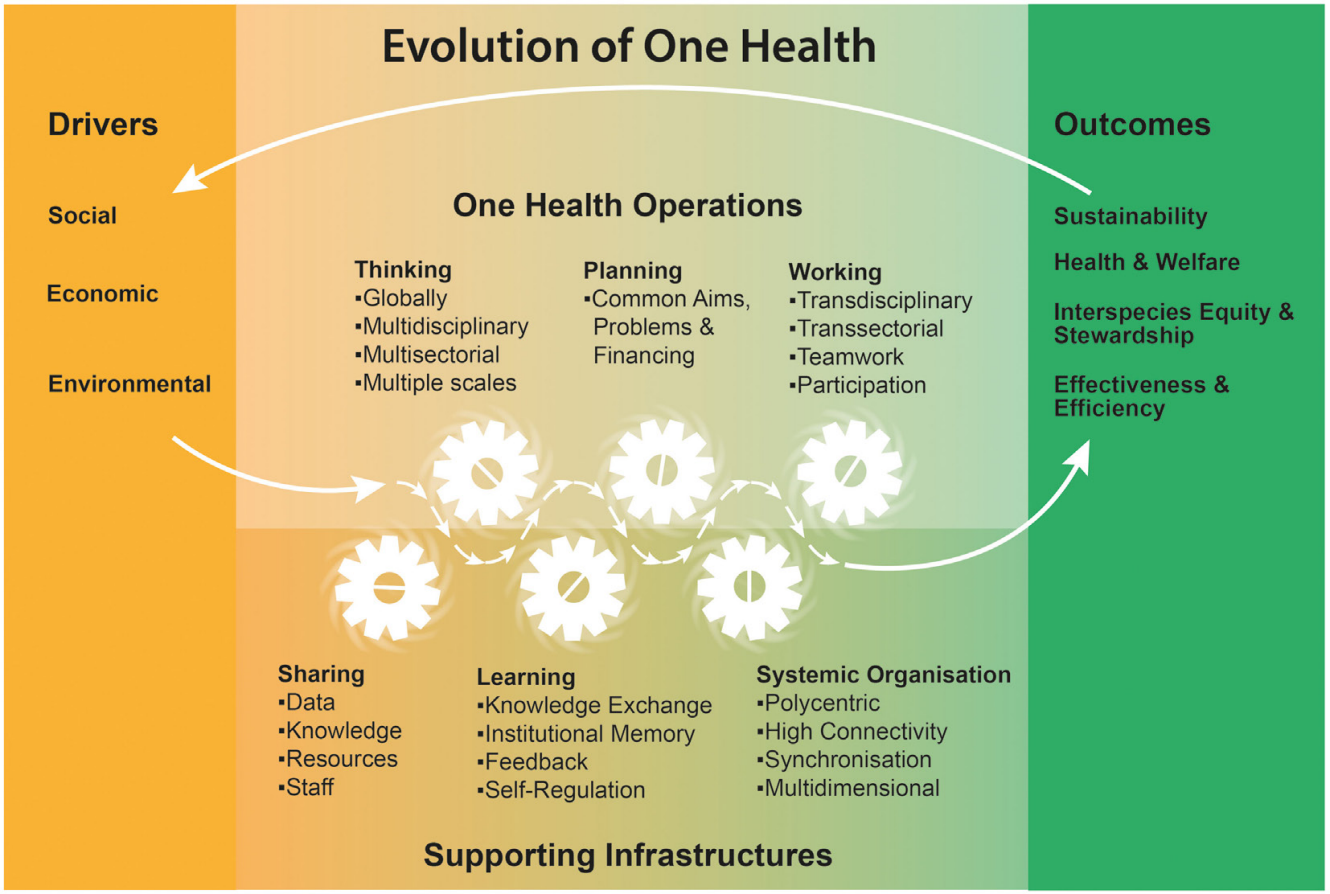
**One Health characteristics identified during a workshop held in Cluj, Romania, June 2015, by members of the Cooperation in Science and Technology Action TD1404: Network for Evaluation of One Health (http://neoh.onehealthglobal.net)**.

The characteristics of OH presented here resulted from a NEOH workshop held in June 2015. Twenty-five experts from 14 countries representing public, human, veterinary, wildlife and environmental health, food safety, agriculture, agro-economics, geography and development aid, research, government, and international organizations attended. The notion evolved that there are specific conditions that demand integrated approaches, which we named drivers. At the other end, specific outcomes are expected to be produced as a result of these integrated OH approaches. The principal OH approach as such was considered to consist of a specific operational paradigm requiring a supporting infrastructure to become effective. Figure 1 illustrates the relations between drivers, operations, supporting infrastructure and outcomes of OH.

### Drivers

Factors identified as drivers (Figure 1) define the need for change toward OH approaches, based on a collective perception of a given problem. Such shared awareness reflects the multiple and complex drivers behind health problems. In reference to the social determinants of health identified by the World Health Organization (WHO) commission (13), social drivers for integrated approaches include lack of participation, cohesion, and welfare, as well as the presence of ignorance, poverty, poor governance, inequality, violence, mental and physical illness, or high risks for these. Environmental drivers include climate change, land degradation, reduced biodiversity, and ecosystem changes rooted in both natural phenomena and human actions. Economic drivers are mostly related to the globalization process, dominated by market deregulation and financial capital, and largely irrespective of social needs at the local level (14, 15). In this context, the capacity of nations to support public health services and welfare has been progressively eroded and the increasingly scarce resources require enhancement of inter-sectoral synergies, establishment of adequate governance structures, and effective achievement of multiple outcomes simultaneously. Human, animal, and plant populations are affected in many different ways by this process, potentially further widening the gap in human’s access to health and welfare. These examples are by no means exhaustive, and there is clearly an interplay between different drivers. For example, globalized trade agreements may lead to land acquisition by large multinational companies, thereby creating land shortages for local populations who are pushed to intensified extraction of available natural resources. Increased poverty in conjunction with close contact to previously unexploited environments puts human and animal health at risk (16). At the same time, economic crises and financial deregulation reduce public resources for interventions, thereby reinforcing negative environmental, economic, and social drivers and exacerbating negative health outcomes (17).

### Operations

Although OH initiatives can range from development projects to educational programs, research projects, and intergovernmental strategies, they often have specific operating principles, characterized by a way of thinking, planning, and working. We selected this classification, as it represents a sequence from abstract thoughts over planning of an initiative to concrete implementation. The realization that certain health and welfare challenges cannot be dealt with from a single disciplinary perspective thus calls for a re-evaluation of approaches to deal with health and welfare challenges. “OH thinking” is holistic, inclusive, respectful, and tolerant, as opposed to approaches that are specific, reductionist, with a tendency to focus on single or limited outcomes that impact positively on few people only. It considers multiple scales of life, disciplines, sectors, species, paradigms, and demographics, and integrates at different spatial scales (e.g., locally, nationally, and globally). This should reflect the connected nature of social relations and social systems, both in their material and symbolic dimensions as well as the degradation of national resources due to globalization (18). “OH planning” requires that aims, problem formulation, responsibilities, and financing are organized, regardless of organizational hierarchies, paradigms, sectors, and disciplines. Most fundamentally, it necessitates clarity in establishing roles, tasks, responsibilities, and competencies (including leadership, power, and authority) within the specific OH initiative. OH aims to identify acceptable and manageable solutions to problems within a given context. Only after establishing a consensus, it (OH) can work and responsibilities be effectively allocated within the system.

### Supporting Infrastructure

Consequently, “OH working” relies on transdisciplinary collaboration that embraces contributions from the biological, natural and social sciences, and actively includes stakeholders in the process, from problem definitions to resolution. To operate as conceived, OH must rely on adequate information infrastructure and foster learning across all scales and fields (19). A learning framework allows for stakeholders and institutions to evolve and improve autonomously, and requires mechanisms for knowledge exchange, institutional memory, feedback, and regulation. This relies on sharing of knowledge, data, resources, and staff across sectors and disciplines. This working paradigm will often lead to complex, polycentric organizational structures that support development toward sustainability and resilience (20). To succeed, they rely on multiple, strong connections and coordinated activities across sectors, for example, joint health services for humans and animals (21, 22), and/or for the environment (23).

### Outcomes

The expected outcomes of OH initiatives are health and welfare of humans, animals, plants, and ecosystems, all managed by common health strategies. This ensures healthy food, as well as clean water and air. Transdisciplinarity should result in improved stewardship and compliance, and promote interspecies equity, which would facilitate sustainable benefits for humans from other species (domestic and wild) and their habitats. Furthermore, OH should improve effectiveness across different sectors and at multiple scales. It relies on and results in more efficient communication, thereby generating a higher degree of awareness that can enable rapid detection of illness and consequent action. By having a more inclusive voice for neglected human populations, animals, and environment, OH is intended to widen our usual anthropocentric perspectives and to simultaneously enhance human health. The expected outcomes of OH approaches contribute to the three pillars of sustainability, namely, society, environment, and economy. In this way, the approach can be an instrument to working toward the UN sustainable development goals.^2^ Overall, OH is expected to result in the consideration of long-term effects of policy decisions, resilience at various scales, food and feed security, and ultimately sustainable lifestyles.

## Discussion

### The Added Value of OH

Most diseases identified by WHO in their global burden of disease analysis,^3^ from neglected zoonotic and tropical diseases to lifestyle diseases (e.g., depression, arthritis, cardiovascular diseases, allergies, and malnutrition) are derived, to some degree, from the social–ecological system in which they occur. Many isolated disciplinary or sectoral approaches to deal with these health challenges have proven ineffective, either not durable or associated with economic and/or environmental damage (4, 7). The change of focus from disease to health across species, ecosystems, and scales constitutes an effective model to address these challenges. This model extends from cells, through individuals, populations to global systems and across different time scales (4). Some propose considering health beyond health, i.e., the global economic, political, and cultural context, where, for example, the changing patterns of emerging diseases in Africa or Asia may be caused by investment strategies at the New York, London, and Hong Kong stock exchanges (17, 24).

As demonstrated by the global AIDS response, this inclusive governance challenges current global norms, calls for global accountability, and reveals inextricable links between health, human rights, and social, economic, and political empowerment (25). By formulating apparently distant threats, such as climate change or soil erosion, from a health perspective, legal or economic actions may be accelerated, thereby leading to political decisions (6), through the willingness to accept trade-offs. Many health decisions are linked to dilemmas between scales, namely, individual versus social or global ecological interests. The solution lies in a continuous process of negotiation that includes all stakeholders and results in benefits from the interaction between different sectors (26). OH acknowledges that people’s choices are made within a context of economic, social, and cultural values.

Such a change in approach requires resources. Consequently, it is important to demonstrate common interests of economic, environmental, social, and health advocates to provide appropriate funding, albeit under challenging economic constraints (6). We identified clear parallels between OH and the concept of sustainability with its three pillars, i.e., society, environment, and economy. On this basis, the added value of OH as compared to single sector approaches can be assessed through monitoring aspects in these three pillars. For example, the social dimension may be monitored by examining the acceptability of interventions, the contribution to enhancing human capital, supporting solidarity, maintaining equity, diversity, participation, interconnectedness and partnerships, democracy, and political alignment and their adaptations to the relevant social–ecological context. The environmental dimension may be monitored using fresh-water quality, ocean quality, air and soil quality, biodiversity, species-specific health, and the overlap with ecosystem services. Finally, the economic dimension can be assessed by estimating the costs and the benefits of interventions to the widest possible extent, including not only the values that can be directly appraised through market prices but also the values of non-market goods and resources, which mostly depend on environmental and social achievements and are of particular importance for human and animal health and welfare.

### The Realization of OH

To achieve systemic and scaled resilience to health challenges, the ultimate task for policy makers and other health professionals is to endorse health of people, animals, plants, and the environment and to achieve equitable and sustainable health outcomes (27, 28). To implement the concept of OH, “OH thinking,” “planning,” and “working” promote equity beyond health services and keep health (human, animal, and environmental) as the central focus (1, 2). Resilience in human and animal population health has clear benefits for the environment and the economy, at both national and global levels. Additionally, maintaining health is more ethical than facilitating recovery from illness (27). In many cases, isolated policies have contributed to ineffective responses to (emerging) infectious and non-communicable diseases. Health is essential for societal well-being, and many current health challenges are beyond the capacity of any one discipline or jurisdiction to meet. We need to embrace this and facilitate appropriate and sustained responses. For example, the emergence of infectious diseases, including zoonoses, and multidrug resistance is determined by variables including economic conditions, population changes (both humans and animals), and land use changes (29, 30). The consequences include changes in behavior and habits, as well as in intensification of production, trade, habitat change, loss of biodiversity, and globalization (24, 31). These in turn affect the economic conditions, population numbers, and land use, which emphasizes the co-evolutionary nature of these interactions. The long history of cohabitation between humans and animals suggests multiple synergistic effects. However, current trends lead to segregation of species in isolated habitats with complete loss of these synergies. The OH concept can shape this cohabitation, in a positive way, rather than being driven by fear and rejection. Ideas such as zoobiquity explore how animal–human commonalities can be used to diagnose, treat, and heal patients of all species, not just humans (32). The current challenge is to shape national, regional, and global institutions to facilitate these transdisciplinary processes and to provide methods to assess their level of integration as well as the evolution of the affected system. Health professionals have previously been at the forefront of social change, gradually making smoking and poor dietary habits increasingly unacceptable (6). It is now time to advocate for continuous adaptation of the underlying determinants of health, in particular interspecies equity, stewardship, and resilience to achieve a healthy and sustainable future for all.

## Author Contributions

The article is the result of a workshop. Writing of the article with equal contribution: SR and BM. Senior author supervising the writing: AL. Organization and moderation of the workshop: BH, AM, and SR. Preparatory presentations for the workshop: BH, CIS, TE, MP, MA, JZ, PD, and AM. Workshop participation and article editing: BH, CIS, MP, MA, JZ, SB, JR, LC, DDM, LRN, MC, MF, FG, VI, DM, HO, MR, RK, and AS.

## Conflict of Interest Statement

The authors declare that the research was conducted in the absence of any commercial or financial relationships that could be construed as a potential conflict of interest.

## Footnotes

^1^http://neoh.onehealthglobal.net.

^2^http://www.un.org/sustainabledevelopment/sustainable-development-goals/.

^3^http://www.who.int/topics/global_burden_of_disease.
